# The Dynamic Roles of TGF-β Signalling in EBV-Associated Cancers

**DOI:** 10.3390/cancers10080247

**Published:** 2018-07-27

**Authors:** Sharmila Velapasamy, Christopher W. Dawson, Lawrence S. Young, Ian C. Paterson, Lee Fah Yap

**Affiliations:** 1 Department of Oral & Craniofacial Sciences, Faculty of Dentistry, University of Malaya, 50603 Kuala Lumpur, Malaysia; vsharmila84@gmail.com (S.V.); ipaterson@um.edu.my (I.C.P); 2 Institute of Cancer and Genomic Medicine, University of Birmingham, Birmingham B15 2TT, UK; c.w.dawson@bham.ac.uk; 3 Warwick Medical School, University of Warwick, Coventry CV4 7AL, UK; L.S.Young@warwick.ac.uk; 4 Oral Cancer Research and Coordinating Centre, University of Malaya, 50603 Kuala Lumpur, Malaysia

**Keywords:** TGF-β signalling, Epstein-Barr virus, nasopharyngeal carcinoma, gastric cancer, B-cell lymphoma

## Abstract

The transforming growth factor-β (TGF-β) signalling pathway plays a critical role in carcinogenesis. It has a biphasic action by initially suppressing tumorigenesis but promoting tumour progression in the later stages of disease. Consequently, the functional outcome of TGF-β signalling is strongly context-dependent and is influenced by various factors including cell, tissue and cancer type. Disruption of this pathway can be caused by various means, including genetic and environmental factors. A number of human viruses have been shown to modulate TGF-β signalling during tumorigenesis. In this review, we describe how this pathway is perturbed in Epstein-Barr virus (EBV)-associated cancers and how EBV interferes with TGF-β signal transduction. The role of TGF-β in regulating the EBV life cycle in tumour cells is also discussed.

## 1. Introduction

The transforming growth factor-beta (TGF-β) superfamily is a group of multifunctional proteins comprising more than 40 members that are clustered in several subfamilies, which include TGF-β, activins/inhibins, bone morphogenetic proteins (BMPs), nodal and growth differentiation factors (GDFs) [1,2]. The prototypic member, TGF-β1, is produced by a diverse range of cell types and modulates cell proliferation, migration, adhesion, differentiation and survival [2,3]. Consequently, a malfunctioning TGF-β pathway is central to many diseases including cancer. TGF-β functions as a tumour suppressor by inhibiting the growth of untransformed epithelial, endothelial and lymphoid cells [4,5,6] and resistance to TGF-β is regarded as one of the crucial steps in malignant progression [2,7]. In the early stages of cancer development, TGF-β signalling functions as a tumour suppressor by inhibiting cell cycle progression from G1 to S phase and inducing apoptosis, senescence and differentiation [2,5,8,9]. Conversely, in late stage disease, it acts as a tumour promoter by inducing epithelial-to-mesenchymal transition (EMT), migration, invasion, metastasis, angiogenesis and immune suppression [2,9,10,11,12]. Frequently, cancer cells become resistant to the tumour suppressive effects of TGF-β, however functional TGF-β signalling often persists in these cells enabling TGF-β-induced tumour promoting phenotypes [13,14,15,16]. Accumulating evidence has revealed that the TGF-β signalling pathway is targeted by many oncogenic viruses, including Epstein-Barr virus (EBV), during the course of tumorigenesis [17]. 

EBV was the first human cancer virus to be discovered [18] and the most common viral infection in humans. Following its discovery in Burkitt lymphoma (BL), EBV has been linked to the aetiology of multiple human cancers of both lymphoid and epithelial origin, including classical Hodgkin lymphoma (HL), diffuse large B cell lymphoma (DLBCL), post-transplant lymphoproliferative disorders (PTLD), nasopharyngeal carcinoma (NPC) and EBV-associated gastric cancer (EBVaGC) [19]. Here, we provide an overview of our current understanding of the dynamic roles that TGF-β plays in EBV-associated malignancies.

## 2. TGF-β Signalling

**Figure 1:**
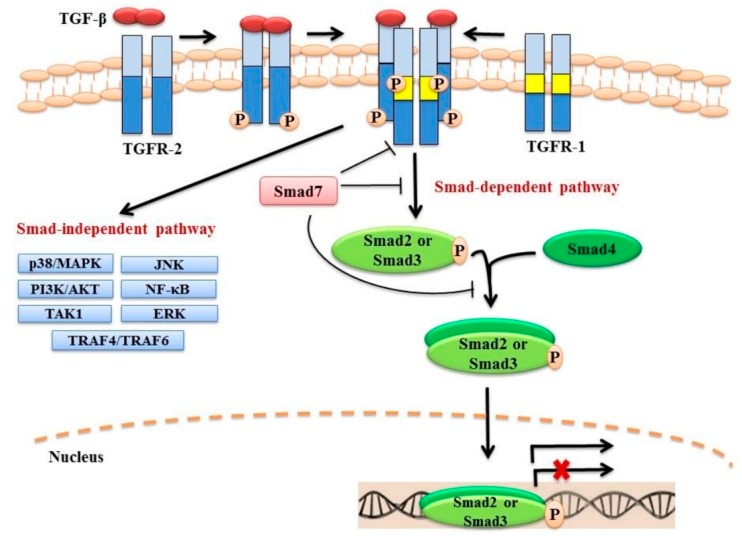
The TGF-β signalling pathway. Binding of an activated TGF-β ligand to TGFR-2 recruits and activates TGFR-1. This, in turn, phosphorylates Smad2 and/or Smad3 (R-Smads), which then form complexes with Smad4 (Co-Smad) and translocate into nucleus to regulate the transcription of various target genes. Smad7 (I-Smad) inhibits the pathway through various mechanisms, including mediating the degradation of TGFR-1, inhibiting phosphorylation of Smad2/Smad3 or inhibiting the formation of the Smad2/3-Samd4 complex. In addition to the canonical Smad-dependent signalling, activated TGF-β receptors can trigger other signalling pathways including ERK-MAPK, p38-MAPK, PI3K-Akt and JNK.

In mammals, there are three TGF-β isoforms (TGF-β1, TGF-β2 and TGF-β3), each encoded by different genes [20,21,22]. These isoforms are highly similar and share approximately 70–80% sequence homology [22,23,24]. TGF-β1 was the first isoform to be characterized and is the most studied to date [20,21]. TGF-β1 is synthesized in a latent form as a large precursor protein which binds to and is stored in the extracellular matrix (ECM) [25]. The precursor protein undergoes proteolytic digestion by the endopeptidase furin to produce two proteins, namely latency-associated peptide (LAP; 278 amino acids) and mature TGF-β1 (112 amino acids) [26,27]. Despite the cleavage of the precursor protein, the LAP remains bound to the mature TGF-β1 making the TGF-β1 biologically inactive [28]. The release of active TGF-β1 from the ECM can be triggered by several factors, such as changes in the cellular environment, tissue injury or inflammation [29,30,31]. Activated TGF ligands mediate signalling through the TGF-β type I and type II receptors (TGFR-1 and TGFR-2, respectively) that are endowed with serine/threonine kinase activity [32,33]. Upon binding of an active TGF-β ligand to TGFR-2, TGFR-1 is recruited and phosphorylated by TGFR-2. The activated heterotetramer TGFR-1/TGFR-2 complex triggers the canonical Smad-dependent, as well as non-canonical Smad-independent signalling pathways (Figure 1). 

### 2.1. Canonical Smad-Dependent Signalling

Smad proteins were the first identified downstream signalling transducers of TGF-β [34]. The proteins of the SMAD family are the vertebrate homologs of the *Drosophila* mothers against decapentaplegic (MAD) protein and the *Caenorhabditis elegans* small body size (SMA) protein [35,36]. These proteins are divided into three groups based on their functions: receptor-activated Smads (R-Smad; Smad2 and Smad3), common mediator Smad (Co-Smad; Smad4) and inhibitory Smads (I-Smads; Smad6 and Smad7) [37,38,39]. Following TGF-β binding to TGFR-2 and receptor activation, the Smad anchor for receptor activation (SARA) protein binds TGFR-1 and Smad2 and/or Smad3 simultaneously, resulting in the phosphorylation of the Smad2 and/or Smad3 by TGFR-1 [40,41,42]. Upon activation, Smad2 and/or Smad3 are released from the receptor complex and SARA, and oligomerize with Smad4 through their MH2 domains [40,42,43]. The Smad2/Smad4 and/or Smad3/Smad4 complexes then translocate to the nucleus to either stimulate or repress the transcription of their target genes, depending on interactions with various transcription factors [44]. A variety of transcription factor families have been identified that act in concert with Smad proteins, including p300/CBP, AP1, and Forkhead [45,46,47].

The two I-Smads, Smad6 and Smad7, tightly control the activation of TGF-β signalling. Compared to Smad6, Smad7 has been shown to inhibit TGF-β signalling more efficiently through a number of mechanisms [48,49]. These include inhibition of the phosphorylation of R-Smads by forming a complex with activated TGFR-1, degradation of the activated TGFR-1 by recruiting ubiquitin E3 ligases, such as Smurf1/2 or disruption of the formation of functional Smad-DNA complexes in the nucleus [50,51,52,53]. Recent evidence also demonstrated that Smad7 might directly oligomerize with R-Smads and inhibit their activities [54]. 

### 2.2. Non-Canonical Smad-Independent Signalling

While the canonical Smad-dependent pathway has been regarded as the major signalling route of TGF-β, the ligand can also signal through non-canonical Smad-independent pathways, engaging the ERK-MAPK, p38-MAPK, PI3K-AKT and JNK pathways [1,2]; different mechanisms are utilised to trigger these signalling pathways. For example, activation of the ERK-MAPK pathway is mediated by the phosphorylation of TGFR-1, whereas the activation of both TGFR-1 and TGFR-2 are required for activation of the PI3K-AKT signalling pathway [55,56,57]. Notably, activation of the canonical Smad-dependent and the non-canonical Smad-independent signalling pathways is not mutually exclusive [58,59]. In breast cancer cells lines, for example, both pathways act together to mediate TGF-β-induced growth arrest [59]. 

## 3. Resistance of EBV-Positive Cells to TGF-β-Mediated Cytostasis 

In normal epithelial and neuronal cells, TGF-β1 inhibits progression from G1 to S phase of the cell cycle by inducing the expression of CDK inhibitors, p15 and p21, thereby blocking the phosphorylation of the Rb protein [60,61,62]. In addition, the increase in p15 levels induces the release of p27 from CDK4 and/or CDK6 [60]. p21 and the free p27 bind to CDK2, inhibiting formation of cyclin A-CDK2 and/or cyclin E-CDK2, thereby blocking the progression to S phase [60,62]. Further, TGF-β1 suppresses the expression of the c-MYC protein, preventing c-MYC from inhibiting the expression of p15, p21 and p27 [63,64,65]. This safeguards the induction of the CDK inhibitors and thereby leads to G1 cell cycle arrest. Additionally, TGF-β1 has been shown to induce both the intrinsic and extrinsic apoptotic programs in a cell-type dependent manner [2]. In lymphoma cells, TGF-β1 induces the intrinsic apoptotic pathway by stimulating the expression of several pro-apoptotic Bcl-2 family members (such as Bmf, Bim and Bax), which in turns suppress the expression of anti-apoptotic proteins (Bcl-XL and Bcl-2) [66]. The ability of TGF-β1 to induce the extrinsic apoptotic program has been shown in liver and lung cancer cells, in which expression of death-associated protein kinase (DAPK) and Fas-mediated apoptosis was increased upon the exogenous addition of TGF-β1, respectively [67,68]. 

EBV-positive and -negative B cells exhibit differential responses to TGF-β. EBV-negative B cells are sensitive to TGF-β-mediated growth inhibition and apoptosis [69,70,71], whilst these responses are lost in EBV-positive B cells [72,73,74]. Similarly, gastric tissue-derived EBV-infected epithelial cell lines (GT38 and GT39) have been shown to be resistant to TGF-β1-mediated growth inhibition and apoptosis, as opposed to a TGF-β1-responsive EBV-negative gastric carcinoma cell line HSC-39 [75]; similar observations were also obtained in our laboratory with the EBV-positive NPC cell line, C666-1 (Yap L.F.; Dawson C.W. (University of Malaya, Kuala Lumpur, Malaysia) Personal observation, 2013). However, the growth of an EBV-negative NPC cell line CNE-2 was not suppressed by exogenous TGF-β1 [76]. CNE-2 cells were originally derived from a poorly differentiated NPC which was likely to be EBV-positive [77]. Although CNE-2 cells do not carry EBV genomes in long-term culture, it is possible that these cells developed resistance towards the cytostatic effect of TGF-β at the initial stage of EBV infection and retained this characteristic even after they lost the EBV genomes. It is reasonable to hypothesize that EBV-infected cells can selectively outgrow the neighboring cells (EBV-negative) which are growth inhibited by TGF-β1 produced by EBV-infected cells through the expression of Zta (discussed below). Such EBV-infected cells can then clonally expand to drive the transformation process. Indeed, it was shown that LMP1-transfected BALB/c 3T3 cells exhibited non-transformed phenotypes in vitro but those that lost sensitivity to TGF-β-mediated growth inhibition formed tumours in severe combined immunodeficiency (SCID) mice [78], implying that loss of TGF-β responsiveness is a critical event for the tumorigenicity of EBV-infected cells.

### 3.1. Contribution of EBV Latent Genes

EBV displays two distinct lifecycles, namely the lytic and latent cycles. The lytic cycle is associated with viral replication in which new virions are produced while latent cycle is a state of persistent infection and the absence of productive viral replication [79]. During latent infection, a limited set of EBV genes is expressed and to date, three latency programmes have been identified. Different types of malignancies are associated with a distinct latency programme (Table 1). The EBV lytic cycle is initiated by the expression of the viral immediate-early gene *BZLF1* (the gene product is commonly known as Zta or ZEBRA) [80]. While the lytic cycle can be triggered in vitro by diverse stimuli such as phorbol ester and sodium butyrate [81], this process is closely associated with the differentiation of both B cells and epithelial cells in vivo [82,83,84].

It has been shown that the EBV oncoprotein LMP1 was responsible for mediating resistance to the cytostatic effects of TGF-β1 in BL cells (BL41) by elevating levels of cyclin D2 [73]. A modest sensitization to TGF-β was observed in EBV-positive lymphoblastoid cell lines (LCLs) following treatment with LMP1 antisense oligodeoxynucleotides [87], although studies of EBV-converted and stably transfected BL cell lines have shown that LMP1 was not sufficient or necessary to block the TGF-β1 response [88]. Using epithelial cells as study models, LMP1 has been shown to abrogate TGF-β signalling through NF-κB-dependent depletion of transcriptional coactivators required for Smad-mediated transcription [89,90]. Additional evidence showed that LMP1 induction of Id1 through suppression of ATF3 (a SMAD-induced transcriptional repressor) attenuated TGF-β-Smad-mediated transcription and counteracted the cytostatic action of TGF-β1 in epithelial cells [91]. The ability of LMP1 to suppress Smad-dependent transcription was also demonstrated in SCC12F epithelial cells [92]. Further, it has been shown that LMP1 can down-regulate the expression of limb-bud and heart (LBH) resulting in the alleviation of TGF-β1-induced NF-kB signalling inhibition, rendering NPC cells refractory to TGF-β1-mediated cytostasis [93]. 

Other EBV-encoded proteins have also been shown to repress TGF-β signalling and this could facilitate the evasion of TGF-β-mediated cytostatic effects during EBV infection. In BL Ramous cells and gastric carcinoma cells (HSC-39), LMP2A has been shown to inhibit TGF-β1-induced apoptosis through the PI3K/AKT pathway [94]. A negative feedback loop between EBNA1 and TGF-β was also postulated, in which expression of EBNA1 in a nasopharyngeal adenocarcinoma cell line, AdAH, has been shown to repress TGF-β1-induced transcription by increasing Smad2 protein turnover [95], an effect that may overcome the ability of TGF-β to repress the Q promoter (Qp), which is responsible for EBNA1 expression in NPC [96]. The strategic inhibition of B-cell apoptosis is central to EBV biology. In germinal centres, only those B-cells that express the highest-affinity immunoglobulins are rescued from stringent pro-apoptotic pathways that signal through TGF-β, FAS and B-cell receptors [97,98,99]. The ability of EBNA1 to disable TGF-β signalling was also demonstrated in HL cells, where EBNA1 increased Smad2 protein degradation which subsequently inhibited transcription of the TGF-β target gene, PTPRK tumour suppressor, contributing to the growth and survival of HL cells [100]. In B-cells exhibiting a group III latency program, EBNA2 antagonized the apoptotic effects of TGF-β1, partly by repressing the pro-apoptotic “sensitizer” protein, BIK, resulting in B-cell survival [101,102]. Further, BARF1 was found to promote gastric cancer cell proliferation through a mechanism involving the downregulation of Smad4 via an increase in NF-κB-dependent miR-146a [103]. Taken together, it is apparent that TGF-β signalling is disrupted by EBV-encoded latent genes by a variety of mechanisms leading to malignant transformation.

### 3.2. Dysregulation of TGF-β Receptors

In order to evade the tumour suppressive effects of TGF-β1, cancer cells often develop genetic abnormalities within key molecules of the TGF-β signalling pathway, particularly the TGF-β receptors, *TGFBR1* and *TGFBR2*. However, the contribution of alterations in TGF-β receptor expression to the loss of responsiveness towards TGF-β1-mediated growth inhibition in EBV-positive cells is inconclusive. While some studies showed that the levels of *TGFBR1* and *TGFBR2* did not correlate with resistance [73,75,78], others reported that the lack of responsiveness appeared to correlate with a down-regulation of *TGFBR2* expression [88,104]. These observations suggest that multiple mechanisms regulate the growth inhibitory response to TGF-β in EBV-positive cells. Nonetheless, alterations in the expression of TGF-β receptors have been shown in EBV-associated cancers in vivo. The down-regulation of TGF-β receptors in cancer cells can be caused by multiple mechanisms. For example, the expression of *TGFBR2* can be reduced by mutation, promoter hypermethylation or miRNA regulation. It is noteworthy that TGFR-2 expression has been suggested as a positive prognostic marker in DLBCL patients [105]. Further, the mRNA and/or protein levels of *TGFBR1* and *TGFBR2* were found to be significantly reduced in primary NPC tissues compared with non-cancerous controls, and their decreased expression correlated with poor survival [106,107,108,109]. However, a recent report described contradictory results in which TGFR-1 was found to be up-regulated in primary NPC tissues [110]. We previously had reported the expression of TGFR-2 in oral cancer by immunohistochemical analysis [111] and accurate staining results could only be achieved by applying stringent methodologies and assessment. The discrepancy between studies could be due to differences in antibody specificities. It is worth noting that *TGFBR2* is located at chromosome 3p, a region with the most frequent loss of heterozygosity in NPC [112,113], implying that *TGFBR2* might be a tumour suppressor gene that is altered in the early stages of NPC pathogenesis. Using advanced next-generation sequencing technology, several studies have reported genetic abnormalities of key molecules within the TGF-β pathway, including the TGF-β receptors, in EBV-associated cancers (Table 2). Although the frequency of the genetic alterations appears to be low, further studies are warranted to confirm the results and investigate the functional significance of these alterations. It is important to recognize, however, that these results do not take into account possible transcriptional alterations of the receptors and/or signalling molecules. 

The involvement of cellular miRNAs in the disruption of TGF-β signalling has also been reported. For example, miR-93 and miR-19a, paralogues of the oncogenic miR-17-92 cluster, were shown to promote NPC aggressiveness by down-regulating TGFR-2 [108,109]. Several studies on global miRNA profiling in NPC have identified a number of differentially expressed miRNAs that target the TGF-β pathway [114,115,116], but the exact targets within the pathway are yet to be identified. Notably, a susceptibility gene TNFRSF19 in NPC, was shown to render NPC cells resistant to TGF-β-mediated cell cycle arrest [117]. TNFRSF19 was highly expressed in NPC and binds specifically to the kinase domain of TGFR-1, thereby blocking Smad2/3 association with TGFR-1 and subsequent signal transduction. 

## 4. Tumour Promoting Roles of TGF-β 

TGF-β1 exerts its tumour promoting effects by inducing EMT, migration, invasion, metastasis, angiogenesis and immune suppression [2,9]. High levels of TGF-β1 are commonly detected in many types of solid tumour and positively correlate with disease stage [130,131,132,133]. TGF-β1 can be produced by tumour cells or by stromal cells in the tumour microenvironment, including macrophages and platelets [134,135]. As tumours progress, many cancer cells develop genetic abnormalities within the pathway to escape the tumour suppressive effects of TGF-β signalling and, therefore, the excessive production of TGF-β1 drives tumour progression [2,7]. Although it is suggested that the tumour promoting effects of TGF-β1 are mainly mediated through the non-canonical Smad-independent signalling pathway [136,137,138,139], there is evidence to show that the canonical Smad-dependent signalling pathway can also be responsible for mediating some of these effects [1,2,3]. 

### 4.1. High Levels of TGF-β in EBV-Associated Cancers 

Several early studies showed that TGF-β1 and TGF-β2 were produced by Hodgkin’s Reed-Sternberg (H-RS) cells in vitro and in vivo [140,141,142,143]. It was subsequently shown that HL cells produced TGF-β, which contributes to the shift from a Th1-biased towards a Th2-biased T-cell infiltrate characteristics of HL [144]. EBV and its lytic gene product (Zta) have been shown to induce TGF-β1 production and secretion in BL and HeLa cells [145,146]. In patients with NPC, the levels of both the total and active TGF-β1 in serum samples have been reported to be elevated compared to those from healthy individuals with levels positively correlating with disease staging [147]. In support of these observations, our previous study has confirmed that TGF-β1 was up-regulated in EBV-positive NPC tissues compared to non-malignant nasopharyngeal mucosa [148]. There is also evidence to show that EBNA1 and LMP1 induced the expression and secretion of TGF-β1 in epithelial cells in vitro [92,149]. Interestingly, a relatively high intracellular expression of TGF-β1 protein was detected following miR-93-mediated down-regulation of *TGFBR2* in NPC cells [108]. In gastric cancer, high levels of TGF-β1 and TGF-β3 expression were detected in tissue samples of gastric carcinoma compared to gastric mucosa, although the status of EBV in these cancer samples examined was unreported [150]. Further, both gastric tissue-derived EBV-infected epithelial cell lines, GT38 and GT39, spontaneously produce biologically active TGF-β1 [75]. These data are consistent with the more recent report that TGF-β1 levels were elevated in EBVaGC [151]. Notably, several mutations on TGF-β1 and TGF-β2 have been detected in EBV-associated cancers (Table 2), pointing to a possible role of EBV in regulating the expression of TGF-β ligands. Further investigations are warranted to examine whether these are gain-of-function mutations that might result in increased levels of TGF-β ligands.

### 4.2. Contribution of TGF-β Signalling to the Aggressive Phenotypes of EBV-Associated Cancers

Several lines of evidence have shown that TGF-β signalling promotes aggressive phenotypes of EBV-associated epithelial cancers. TGF-β signalling is a major inducer of EMT in cancer cells [152]. EMT is morphologically characterized by changes from an epithelial cell phenotype to a spindle fibroblast-like appearance and functionally characterized by decreased cell adhesion and increase cell migration. Accordingly, TGF-β signalling-associated induction of EMT is considered an important step in the progression of tumour metastasis. Alterations in EMT markers (increased Vimentin and decreased E-cadherin) were detected in clinical NPC samples [153,154], indicating that NPC cells undergo EMT in vivo. Although EBNA1 has been shown to suppress TGF-β-mediated transcription in AdAH and HL cells [95,100], in NPC cells, EBNA1 appeared to up-regulate the expression of TGF-β1 protein leading to a reduction in expression of miR-200a and miR-200b which in turn, up-regulated their target genes ZEB1 and ZEB2, well known mediators of EMT [149]. Recent reports have repeatedly described the underlying mechanisms of the EMT process induced by TGF-β signalling in NPC, and a number of effectors have been identified. There is evidence to demonstrate that components of lipid rafts, flotillin-1 and -2 (Flot1 and Flot2), were highly expressed in primary NPC tissues [155,156] and that this contributed to the TGF-β1 induction of EMT in NPC. Flot1 was shown to stimulate the expression and secretion of TGF-β1, facilitate the activation of TGF-β/Smad3 signalling to effectuate EMT in NPC cells [155]. Whereas Zhao and colleagues showed that Flot2 was required for TGF-β1-induced EMT in NPC cells through activation of Src [156]. Further, the ability of high-mobility group AT-hook 2 (HMGA2) to induce EMT in NPC cells was attributed to the activation of TGF-β/Smad3 signalling pathway [157]. TGF-β1 was also shown to induce EMT in NPC cells by enhancing the expression of formin-like 3 (FMNL3) and Y-box binding protein-1 (YBX1) [153,154]. More recently, it was found that TGF-β1 induced NPC cell growth and migration by down-regulating miRNA-124 which inhibited TGF-β1-mediated responses by targeting the pro-oncogenic lncRNA MALAT1 primarily via the ERK/MAPK pathway [158]. In addition, LMP1-mediated activin/ TGF-β signalling through the JNK/SAPK pathway was also involved in the induction of the extracellular matrix protein, fibronectin, a process that may contribute to tumour invasiveness in NPC [92].

In addition to promoting aggressive phenotypes of cancer cells, an emerging role for TGF-β signalling in cancer drug resistance has also been proposed [159,160]. Very recently, it was reported that overexpression of miR-449b in NPC down-regulated TGF-β-induced (TGFβI), a target gene of TGF-β pathway, leading to increased pro-TGF-β1 activation and cisplatin resistance [161]. The effect of TGF-β in inducing aggressive phenotypes in EBVaGC is currently unexplored and further studies are warranted.

## 5. Induction of EBV Lytic Reactivation by TGF-β 

EBV is able to induce its lytic cycle by switching on the expression of *BZLF1* gene which encodes protein Zta [80]. TGF-β was initially shown to induce the viral productive cycle in marmoset B lymphocytes immortalized with EBV [162]. It was subsequently shown that TGF-β induces latent EBV to enter into lytic cycle (as shown by EA expression) in two BL cell lines P3HR-1 and Akata [145,163]. These observations were later confirmed in a series of BL cell lines (Mutu-I, Raji and B95-8) in which TGF-β1 induced *BZLF1*/Zta expression by an indirect mechanism which required the ERK 1/2 MAPK kinase pathway; Smad signalling alone was not sufficient to mediate TGF-β1 induction of Zta [164]. It was further shown in additional BL cells (Mutu-I, Kem-I and Sav-I) that the PI3K/AKT pathway, acting downstream of ERK 1/2, enabled Smad3 to be acetylated by direct interaction with the co-activator CREB-binding protein to stimulate TGF-β1-induced Zta expression [165]. Different mechanisms of TGF-β1-mediated activation of *BZLF1* gene have also been reported. In BL cell lines, Rael and P3HR-1, *BZLF1* gene expression appeared to be activated by TGF-β through its mediator Smad proteins [166]. A Smad4-binding element (later termed SBE1) located within the *BZLF1* Z promoter (Zp) was identified and both SBE1 and AP-1 motifs were required for TGF-β to activate the expression of *BZLF1* through the complex of Smad3/Smad4 associated with the c-Jun/c-Fos proteins of the AP-1 complex [166]. However, this mechanism accounted for only 20–30% of the total TGF-β-mediated activation of transcription from Zp. Subsequently, Iempridee and colleagues identified an additional four SBEs (termed SBE2-5) and showed that TGF-β induced EBV lytic reactivation via the canonical Smad pathway by alleviating ZEB-mediated repression of Zp through multiple SBEs acting in concert [167]. In epithelial cells, TGF-β1 partially induced EBV reactivation in gastric cancer cells GT38 and GT39, as shown by the expression of *BZLF1*/Zta and early antigen-D, possibly primarily through junB pathway [75]. These studies have collectively demonstrated that TGF-β induces lytic reactivation in latently EBV-infected cells by stimulating the expression of *BZLF1* gene/Zta protein through both canonical and non-canonical pathways. 

In addition to the *BZLF1* Zp, the *EBNA1* Qp is also a direct target of the TGF-β signaling pathway. EBNA1 is a DNA-binding protein that binds to the ori-P region of the EBV genome and allows the viral genome to be present as an episome in infected cells [168]. Transcription of the *EBNA1* gene in BL and NPC cells is initiated from the Qp [169]. Qp expression is subject to regulation by a number of mechanisms and interestingly, in BL cells, it has been shown that TGF-β transcriptionally repressed *EBNA1* Qp through cooperativity of a Smad3/Smad4 complex and the transcriptional repressor TGIF at the SBE site within Qp [96]. It has also been shown that the Zta protein interfered with JAK/STAT activation of Qp [170] and induced TGF-β production in HeLa cells [146]. Thus, the autocrine/paracrine function of TGF-β is generated by up-regulation of Zta, which then activates the transcription of TGF-β, thereby forming a positive feedback loop to initiate the EBV lytic cycle. In parallel, both TGF-β and Zta repressed Qp to disrupt EBV latency.

## 6. Concluding Remarks

**Figure 2:**
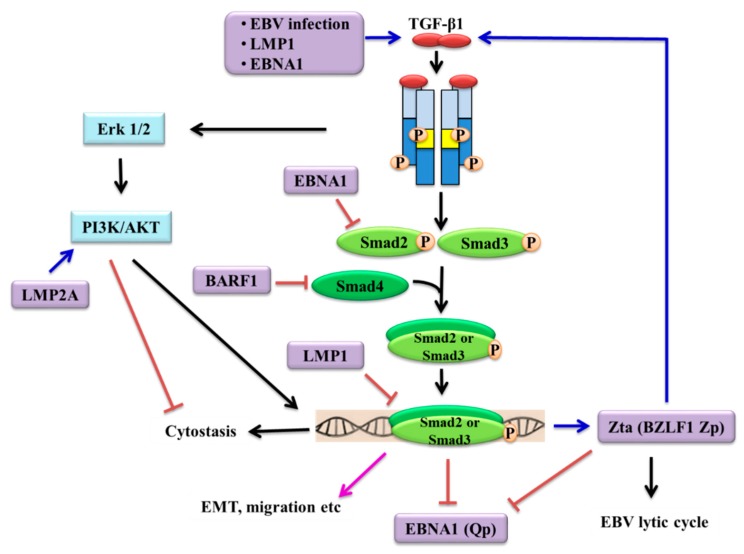
Modulation of TGF-β signalling by EBV. EBV infection or EBV-encoded latent proteins (LMP1 and EBNA1) can stimulate the expression and secretion of TGF-β1 in epithelial cells. However, cancer cells often do not respond to the cytostatic effects of TGF-β, partly through the repression of signal transduction by the EBV-encoded proteins (EBNA1, LMP1, LMP2A and BARF1) through various mechanisms. The cancer cells often sustain a functional TGF-β core machinery and the excessive production of TGF-β drives aggressive malignant phenotypes. TGF-β signalling also appears to be crucial in regulating the balance between latent and lytic cycles in EBV-infected cells. TGF-β facilitates lytic reactivation in EBV-infected cells by stimulating the expression of *BZLF1*/Zta via both Smad-dependent and Smad-independent pathways. Zta induces the production of TGF-β1 which in turn, together with Zta, suppress the transcription of *EBNA1* from Qp to disrupt EBV latency.

Since its discovery in 1964, EBV has been implicated in the aetiology of several tumours of both lymphoid and epithelial origin. Although the mechanisms of EBV infection in lymphoid and epithelial cells are different, it is well-recognised that the virus drives cancer development by de-regulating a diverse range of signalling pathways that regulate essential cellular processes [19]. It is perhaps not surprising that EBV acts as a modulator of the TGF-β signalling pathway, a key network that controls various vital processes, such as proliferation, differentiation, apoptosis and migration. Like many other tumours, EBV-associated cancers produce high levels of TGF-β and do not respond to the cytostatic effects of TGF-β, but yet often sustain a functional TGF-β core machinery to promote more aggressive malignant phenotypes. EBV utilises different mechanisms to manipulate the “double-edged sword” nature of TGF-β signalling to fine-tune the TGF-β response at various levels (Figure 2). A balance between latent and lytic infection is crucial for EBV oncogenesis. In latently infected cells, EBV-encoded proteins (EBNA1, LMP1, LMP2A and BARF1) suppress TGF-β-mediated transcription, rendering cells refractory to TGF-β cytostatic effects. When lytic cycle is needed for the spreading of the virus, TGF-β disrupts the latency by stimulating the expression of *BZLF1*/Zta via both Smad-dependent and Smad-independent pathways which in turn, promotes the production of TGF-β and inhibits the transcription of *EBNA1*. Intriguingly, it has been shown that the EBV infection rate of epithelial cells can be enhanced by exogenous TGF-β1 and TGF-β1 derived from the epithelial cells facilitated viral transmission by inducing lytic cycle in the donor B-cells in co-culture systems [171,172]. These observations imply that TGF-β signalling might play a critical role in regulating persistent EBV infection, particularly in epithelial cells. Several lines of evidence have shown that the expression of *TGFBR2* is down-regulated in NPC and TGF-β/Smad signalling is defective [76,106,107,108,173] and mutations in *SMAD* genes have been reported (Table 2). It is currently unclear whether these defects in the canonical pathway would be adequate to result in the loss of *BZLF1*/Zta expression, thereby facilitate the maintenance of EBV genomes in the nasopharyngeal epithelial cells. This is also relevant to the ability of TGF-β in the induction of differentiation of epithelial cells [174,175]. EBV infection is intimately associated with a number of undifferentiated carcinomas [86], implying that undifferentiated properties of epithelial cells are likely to be a prerequisite for stable EBV latent infection. It has been shown that differentiation of epithelial and B cells triggered EBV lytic reactivation in the latently infected cells [176]. In line with this concept, defects in TGF-β signalling might impair cellular differentiation which results in the suppression of lytic cycle, thereby facilitating latent infection in epithelial cells. Interestingly, it has been shown that in patients with EBVaGC, TGF-β1 levels were significantly associated with the expression of EBV lytic genes in the absence of *Helicobacter pylori* (*H. pylori*) infection [151]. These data imply that *H. pylori* infection prevents EBV lytic induction by suppressing TGF-β1 expression in EBVaGC patients, observations that warrant further investigation. 

While most of the studies on the TGF-β pathway to date have focused on the roles of canonical Smad2/3/4-dependent signalling, emerging evidence has revealed the contribution of non-canonical Smad1/5/9 signalling dysregulation to lymphomagenesis. In DLBCL, oncogenic miR-155 inhibited Smad5 expression and rendered cells resistant to the growth inhibitory effects of both TGF-β1 and BMPs, via a defective p21 induction and decreased formation of the RB/E2F1 complex [177,178]. More recently, Stelling and colleagues identified TGF-β/TGFR-2/Smad1 axis as the upstream regulator in suppressing the expression of sphingosine-1-phosphate (S1P) receptor 2 (S1PR2), a bona fide tumour suppressor in DLBCL, to provide a significant proliferative advantage to DLBCL cells in vitro and in vivo [179]. Interestingly, we have previously reported that EBV infection contributed to aberrant S1P signalling in NPC [180] and also have data showing that BMP signalling is de-regulated in NPC (manuscript in preparation). It will be intriguing to unravel the contribution of EBV infection to the non-canonical Smad TGF-β signalling in the development of EBV-associated cancers. A better understanding of these mechanisms may also provide an explanation for the seemingly contradicting roles of EBV-encoded latent proteins in regulating the TGF-β pathway. EBNA1 and LMP1 have been shown to stimulate the production of TGF-β, and yet, they disrupted the signal transduction rendering the cells refractory to the TGF-β-mediated cytostasis. It is now clear that the high TGF-β production promotes aggressive phenotypes through the EMT induction arm; however, the contribution of EBV to this process is not well-explored. Additional mechanistic studies are warranted to elucidate how EBV fine-tunes the response to TGF-β and utilises this pathway to achieve malignant transformation. Further, it has been shown that TGF-β-induced EMT can drive tumour cells towards a more stem cell-like phenotype [181,182]. Significantly, a decrease in the number of stem cells was observed after treatment with *TGFBR1* inhibitors in gliobastoma [183]. This would be an important research area to be explored in EBV-associated cancers, particularly as EBV latent proteins (LMP1 and LMP2A) have been shown to contribute to the induction and maintenance of cancer stem-like cell (CSC) population in NPC [184,185].

Many drugs that target TGF-β signalling have been developed for the treatment of a number of diseases [186]. Some of which have reached clinical trials, including a phase I trial for EBV-positive lymphoma using recombinant cytotoxic-T-lymphocytes with a virus encoding a dominant negative *TGFBR2* [187]; however, the results have not been posted for this trial. The main goal of utilising anti-TGF-β therapies in cancer is to reduce excessive levels of TGF-β ligands. However, there are clearly concerns and issues with this approach, such as that TGF-β inhibitors are not cytotoxic and might disrupt the stem cells niche resulting in releasing cancer stem cells from dormancy [186]. TGF-β action is highly context-dependent and influenced by multiple factors, such as interactions with other signalling pathways, disease stage and innate genetic background among individuals. EBV infection is likely to add another level of complexity to anti-TGF-β therapies in EBV-associated cancers. It is also noteworthy that the function of TGF-β signalling in the tumour immune microenvironment (TIME) is important in this regard. TGF-β signalling is a crucial mediator not only of changes to the tumour cell phenotype but also of changes in the stromal environment [7]. This is particularly relevant to the development of EBV-based immunotherapies. It has recently been shown that a transcriptional signature of TGF-β pathway activation was associated with low levels of stromal tumour-infiltrating lymphocytes (TILs) and poor prognosis in NPC patients [188]. The TIME in both NPC and HL may influence the response to immunotherapeutic interventions and it might be important to determine the level of TGF-β expression in these tumours as a prognostic indicator of response to such therapies. Therefore, a more complete understanding of the multifaceted function of TGF-β signalling in EBV-associated cancers is required to determine if this pathway can be manipulated therapeutically for the management of patients with these diseases. 

## Figures and Tables

**Table 1:** Characteristics of Epstein-Barr virus (EBV)-associated cancers.

Malignancy	%EBV+ Cases	Latency	EBV Latent Genes
Endemic Burkitt Lymphoma	100%	I	EBNA1, EBER1, EBER2, BARTs, miR-BARTs
Sporadic Burkitt Lymphoma	10–85%		
HIV-associated Burkitt Lymphoma	30–40%		
T/NK cell lymphoma	100%	II	EBNA1, LMP1, LMP2A, EBER1, EBER2, BARTs, miR-BARTs
Hodgkin’s Lymphoma	80–90%		
Hodgkin’s Lymphoma (Nodular sclerosing)	15–20%		
HIV-associated Hodgkin’s Lymphoma	<90%		
Diffuse large B-cell lymphoma (Pythorax lymphoma)	100%	II/III	EBNA1, LMP1, LMP2A, EBER1, EBER2, BARTs, miR-BARTs and/or EBNA2, 3A, 3B, 3C, LP
Diffuse large B-cell lymphoma (in Elderly patients)	>50%		
Diffuse large B-cell lymphoma (late post-transplant)	>50%		
HIV-associated diffuse large B-cell lymphoma	30%		
Post-transplant B-lymphoproliferative disorder	100%	III	EBNA 1, 2, 3A, 3B, 3C, LP, LMP1, LMP2A, LMP2B, EBER1, EBER2, BARTs, miRNAs-BARTs, BHRF1
HIV-associated B-lymphoproliferative disease	100%		
Nasopharyngeal carcinoma	98%	II	EBNA1, LMP1, LMP2A, EBER1, EBER2, BARTs, miR-BARTs, BARF1
EBV-associated gastric cancer	10%		

Epstein-Barr nuclear antigens (EBNA), Latent membrane proteins (LMP), Epstein-Barr virus-encoded RNAs (EBERs), BamH1-A fragment transcripts (BARTs), BamH1 fragment H rightward open reading frame 1 (BHRF1), BamH1 fragment A rightward open reading frame 1 (BARF1). Table adapted from Rickinson [85] and Yap & Lo [86].

**Table 2:** Genetic alterations of TGF-β pathway components in EBV-associated cancers identified by next-generation sequencing.

Cancer	Total Number of Cases	EBV Status	Genes	Alterations	Number of Cases with Alterations	References
NPC	56 primary tumours	Positive	*SMAD3*	Missense mutation	1 (primary tumour)	[118]
NPC	51 primary tumours 8 recurrent tumours 3 local metastatic tumours	Positive	*TGF-β1*	Missense mutation	1 (primary tumour)	[119]
			*TGF-β2*	Missense mutation	1 (primary tumour)	
			*TGFBR2*	Missense mutation	1 (primary tumour)	
NPC	78 primary tumours 11 local recurrent tumours 22 distant metastatic tumours	Positive	*TGF-β1*	Missense mutation	1 (primary tumour)	[120]
			*TGF-β1*	Nonsense mutation	1 (local recurrent tumour)	
			*TGF-β1*	Silent mutation	1 (primary tumour)	
			*TGF-β2*	Frame shift deletion	1 (local recurrent tumour)	
			*TGF-β2*	Inversion	1 (primary tumour)	
			*TGFBR1*	Missense mutation	1 (primary tumour) 1 (local recurrent tumour)	
			*TGFBR2*	Inter chromosomal translocation	1 (primary tumour)	
			*SMAD3*	Silent mutation	1 (local recurrent tumour)	
			*SMAD4*	Missense mutation	1 (primary tumour)	
			*SMAD4*	Nonsense mutation	1 (primary tumour)	
			*SMAD7*	Missense mutation	1 (local recurrent tumour)	
EBVaGC	134 primary tumours	Positive: n = 34 Negative: n = 100	*TGFBR1*	Nonsynonymous mutation	9 (EBV-positive) 8 (EBV-negative)	[121]
	AGS cell line	Before and after EBV infection		Missense mutation	EBV-infected AGS cells	
EBVaGC	22 primary tumours	Positive	*SMAD4*	Missense mutation	2	[122]
HL	7 cell lines	Positive: L591 Negative: SUPHD1, L540, L428, L1236, KMH2, DEV	*SMAD9*	Missense mutation	1 (KMH2)	[123]
HL	5 cell lines	Negative (HDML2, KMH2, UH01, L540, L428)	*TGF-β1*	Amplification	2 (L540, L428)	[124]
			*TGF-β2*	Amplification	3 (KMH2, L540, L428)	
				Deletion	1 (UH01)	
			*TGFBR2*	Amplification	3 (KMH2, L540, L428)	
			*TGFBR3*	Amplification	2 (KMH2, L428)	
			*SMAD1*	Amplification	3 (KMH2, L540, L428)	
				Deletion	2 (HDML2, UH01)	
			*SMAD5*	Amplification	3 (KMH2, L540, L428)	
DLBCL	73 primary tumours 21 DLBCL cell lines	Unreported	*TGF-β1*	Missense mutation	1 (primary tumours)	[125]
			*TGF-β1*	Intronic mutation	1 (primary tumours)	
			*TGFBR2*	Intronic mutation	2 (primary tumours)	
			*TGFBR3*	Intronic mutation	2 (primary tumours)	
			*SMAD9*	Intronic mutation	1 (primary tumours)	
DLBCL	51 primary tumours & immunochemotherapy-treated tumours	Unreported	*TGF-β1*	CNA	3 (treated tumours)	[126]
DLBCL	6 refractory & 7 responsive tumours to R-Chop	Negative	*TGFBR2*	Missense mutation	1 (refractory tumour)	[127]
DLBCL	47 relapsed/refractory tumours 65 primary tumours	Unreported	*TGFBR2*	Missense mutation	6 (relapsed/refractory tumours)	[128]
DLBCL	295 activated B-cell like DLBCL (ABC) 164 germinal-center B-cell like DLBCL (GCB) 115 unclassified DLBCL	Unreported	*TGF-β1*	Missense mutation	5 (4 ABC, 1 GCB)	[129]
				Truncated mutation	2 (ABC)	
			*TGF-β2*	Truncated mutation	2 (1 ABC, 1 GCB)	
			*TGF-β3*	Missense mutation	1 (ABC)	
			*TGFBR1*	Missense mutation	1 (GCB)	
			*TGFBR2*	Missense mutation	2 (1 ABC, 1 GCB)	
				Truncated mutation	2 (1 GCB, 1 unclassified)	
			*TGFBR3*	Missense mutation	2 (1 ABC, 1 unclassified)	
				Truncated mutation	1 (GCB)	
			*SMAD1*	Missense mutation	1 (ABC)	
				Truncated mutation	1 (GCB)	
			*SMAD2*	Missense mutation	3 (1 ABC, 1 GCB, 1 unclassified)	
			*SMAD4*	Missense mutation	3 (2 ABC, 1 unclassified)	
				Truncated mutation	1 (ABC)	
			*SMAD5*	Missense mutation	5 (4 ABC, 1 GCB)	
			*SMAD6*	Missense mutation	1 (GCB)	
			*SMAD7*	Missense mutation	2 (1 ABC, 1 GCB)	
			*SMAD9*	Truncated mutation	1 (ABC)	
